# Area Socioeconomic Status is Associated with Refusal of Recommended Surgery in Patients with Metastatic Bone and Joint Disease

**DOI:** 10.1245/s10434-024-15299-5

**Published:** 2024-06-11

**Authors:** Kyle Mani, Emily Kleinbart, Anne Schlumprecht, Regina Golding, Noel Akioyamen, Hyun Song, Rafael De La Garza Ramos, Ananth Eleswarapu, Rui Yang, David Geller, Bang Hoang, Reza Yassari, Mitchell S. Fourman

**Affiliations:** 1 Albert Einstein College of Medicine, Bronx, NY USA; 2 Department of Neurological Surgery, Montefiore Einstein, Bronx, NY USA; 3 Department of Orthopedic Surgery, Montefiore Einstein, Montefiore Medical Center, Bronx, NY USA

**Keywords:** Bone, Metastases, Socioeconomic status, Race, Surgery

## Abstract

**Background:**

This study sought to identify associations between the Yost Index, a geocoded area neighborhood socioeconomic status (nSES) score, and race/ethnicity with patient refusal of recommended surgery for metastatic bone disease.

**Methods:**

Patients with metastatic bone disease were extracted from the Surveillance, Epidemiology, and End Results database. The Yost Index was geocoded using factor analysis and categorized into quintiles using census tract-level American Community Service (ACS) 5-year estimates and seven nSES measures. Multivariable logistic regression models calculated odds ratios (ORs) of refusal of recommended surgery and 95% confidence intervals (CIs), adjusting for clinical covariates.

**Results:**

A total of 138,257 patients were included, of which 14,943 (10.8%) were recommended for surgical resection. Patients in the lowest nSES quintile had 57% higher odds of refusing surgical treatment than those in the highest quintile (aOR = 1.57, 95% CI 1.30–1.91, *p* < 0.001). Patients in the lowest nSES quintile also had a 31.2% higher age-adjusted incidence rate of not being recommended for surgery compared with those in the highest quintile (186.4 vs. 142.1 per 1 million, *p* < 0.001). Black patients had 34% higher odds of refusing treatment compared with White patients (aOR = 1.34, 95% CI 1.14–1.58, *p* = 0.003). Advanced age, unmarried status, and patients with aggressive cancer subtypes were associated with higher odds of refusing surgery (*p* < 0.001).

**Conclusions:**

nSES and race/ethnicity are independent predictors of a patient refusing surgery for metastatic cancer to bone, even after adjusting for various clinical covariates. Effective strategies for addressing these inequalities and improving the access and quality of care of patients with a lower nSES and minority backgrounds are needed.

An average of 350,000 people die from metastatic disease with osseous extension each year.^1^ Patients who suffer from bone metastases have an increased risk of fracture, bone pain, spinal cord compression, and the need for radiotherapy among other eventualities. In 2022, the National Cancer Institute (NCI) recognized these patients with metastatic cancer as a frequently overlooked subpopulation of cancer survivors who often face burdensome physical and psychological symptoms and have complex care needs.^2^

Previous research has highlighted persistent socioeconomic and insurance-related disparities in the outcomes of ethnic and racial minorities living with metastatic bone disease.^3–5^ The exact causes of these disparities are complex and multifactorial, with patient, provider, and system-related factors playing a role.^6–12^ Understanding the factors that impact the decision of when to receive care, when to accept care, and how to deal with end-stage disease, such as metastatic cancer remains crucial.^13^ Within this context, one critical aspect that warrants closer examination is the refusal of recommended surgery.^14^ The decision to accept or decline surgical interventions can be influenced by a myriad of factors, many of which are intertwined with a patient's nSES.^14^ While previous studies have studied the refusal of recommended surgical intervention in patients with cancers of the colon, head and neck, breast, pancreas, and non-small cell lung cancer, no studies have specifically examined the association between nSES/racial differences in refusal of recommended surgery for patients living with metastatic bone disease.^14–21^

This work examined nSES by using the Yost Index^22,23^—a composite score that is geocoded using census tract-level American Community Service (ACS) 5-year estimates and seven primary measures: median household income, median house value, median rent, percent below 150% of poverty line, Education Index, percent working class, and percent unemployed. The purposes of this work are (1) to determine whether there is a variation in the demographics or characteristics of patients with metastatic cancer to the bones and joints based on the rationale for not receiving cancer-directed surgery and (2) to characterize the association between race/ethnicity and Yost Index with patient refusal of surgical intervention among individuals living with metastatic cancer to the bones and joints. Results may permit the development of interventions to improve access and quality of care for patients from lower nSES backgrounds.

## Methods

### Study Design and Participants

**Fig. 1:**
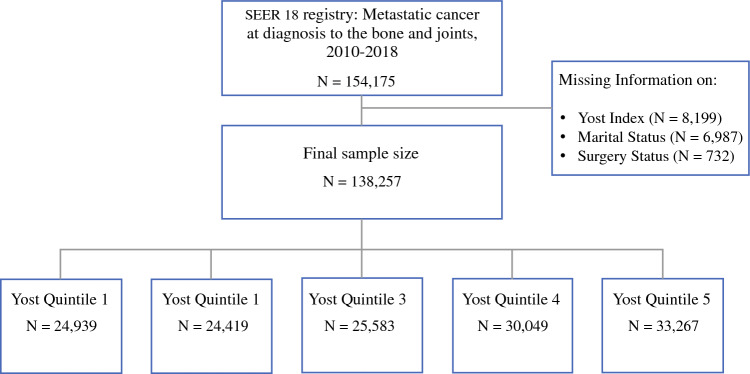
STROBE flow diagram of the Inclusion/Exclusion criteria of the SEER study sample, 2010–2018, stratified by Yost Index Quintile

This was a retrospective, cross-sectional analysis of the population-based data of individuals diagnosed with metastatic cancer to the bone and joints between 2010 and 2018. Patient data were extracted from the SEER 18 database, a network of population-based cancer registries that includes geographic regions of the United States that covers approximately 28% of the population. The SEER 18 database provides information on cancer incidence, survival, and treatment modalities, such as radiation therapy, surgery, and chemotherapy. Of note, the SEER registry does not include information on comorbidities, performance status, surgical pathology, margin status, doses, or agents. To identify patients with bone and joint metastases, we utilized the comprehensive set of International Classification of Disease (ICD-10) codes listed in Table 1. Patients who were diagnosed solely via autopsy or death certificate were excluded from this analysis. The STROBE flow diagram in Fig. 1 illustrates the criteria used for participant inclusion and exclusion in the study.

**Table 1:** ICD-0-3 Site Recodes for the bone and joints

Site description	ICD-O-3 primary site
Long bones of upper limb, scapula, and associated joints	C400
Short bones of upper limb and associated joints	C401
Long bones of lower limb and associated joints	C402
Short bones of lower limb and associated joints	C403
Overlapping lesion of bones, joints, and articular cartilage of limbs	C403
Bone of limb, NOS	C408
Bones of skull and face and associated joints (excludes mandible)	C409
Mandible	C410
Vertebral column	C411
Rib, sternum, clavicle, and associated joints	C413
Pelvic bones, sacrum, coccyx, and associated joints	C419
Overlapping lesion of bones, joints, and articular cartilage	C419
Bone, NOS	C400

"Incidence—SEER Research Plus Data (Specialized with Census Tract SES/Rurality), 18 Regs (excluding AK), Nov 2020 Sub (2010–2018)" was used

### Yost Index and nSES Quintiles

The Yost Index is an effective measure of socioeconomic vulnerability, as validated in previous cancer research studies.^24–26^ The index’s quintiles were calculated via a two-step process using census tract-level American Community Service (ACS) 5-year estimates that were standardized across the entire United States. The first step involved a factor analysis using the following seven variables representing different aspects of nSES: Education (a weighted index that incorporates educational achievement levels, encompassing less than high school completion, high school diplomas, and college degrees), Percent below 150% of poverty line, Percent working class, Percent unemployed, Median rent, Median house value, and Median household income. The second step classified census tracts into nSES quintiles based on equal populations across the United States. The lowest nSES group (first quintile) corresponds with the 20th percentile or lower, whereas the highest nSES group (fifth quintile) corresponds with the 80th percentile or higher. These nSES quintiles were then matched to tumor cases at the census tract level by aligning the ACS survey year with the tumor diagnosis year.

### Statistical Analysis

SEER*Stat 8.4.126 and RStudio version 1.1.383 were used to perform statistical analyses. *Objective 1* involved summarizing patient demographics by age at diagnosis, sex, and race/ethnicity. Chi-squared and analysis of variance (ANOVA) were utilized to assess whether any significant differences existed in the basic clinical covariates between varying reasons for not undergoing cancer surgery. O*bjective 2* used multivariable logistic regression models, with adjusted odds ratios (aORs) with 95% CIs calculated using the exact method. This was undertaken to identify prognostic factors linked to the refusal of surgery, where aOR <1 was deemed a favorable prognostic factor for undergoing surgery and aOR >1 a poor prognostic factor for undergoing surgery. The primary outcome of interest was undergoing recommended surgery of the primary histological site, which was categorized as (1) recommended and performed or (2) recommended but refused by the patient. The key predictor variables were nSES (Yost Index quintile) and race, which was categorized as White, Black, Asian/Pacific Islander (API), or American Indian/Alaskan Native (AI/AN). Other covariates that were included as potential confounders included age, sex, marital status, urban/metropolitan recode, and primary cancer site. Age-adjusted incidence rates were also calculated for the various reasons for either undergoing or not undergoing surgical treatment and were independently stratified by race and nSES. Age-adjusted incidence rates were calculated and standardized to the 2000 U.S. Standard Population (Census P25-1130). Statistical significance was determined by using the Wald test. All hypotheses were one-sided, and *P* < 0.05 was regarded as significant.

## Results

### Patient Demographics and Characteristics

A total of 138,257 patients (2010–2018) with de novo metastatic cancer to the bone and joints from the top ten prevalent primary cancer sites were abstracted from SEER. Of these, 14,943 (10.8%) of these patients had some sort of surgery recommended. Mean age was 67.7 (SD = 12.2), and the majority of patients were male (58.8%, *n* = 81,759), White (78.8%, *n* = 109,585), married (53.8%, *n* = 74,778), and living in a metropolitan area (87.2%, *n* = 121,190). Table 2 shows baseline covariates among the study patient population, stratified by reasons for not undergoing cancer-directed surgical resection. Of all of the patients who had surgery recommended, 19.6% refused. Reasons for this were unknown (9.8%, *n* = 1469), active surgical refusal (7.9%, *n* = 1178), and mortality before surgery (1.9%, *n* = 282). Among those patients who refused surgery, 55.9% were female (*n* = 659) and 64.4% were single (*n* = 759). Surgery was most recommended and performed in patients whose primary cancers were breast (35.8%, *n* = 4302) and prostate (20.5%, *n* = 2459).

**Table 2:** Baseline covariates of patients living with metastatic cancer to the bone and joints, stratified by reason for no cancer-directed surgical resection, 2010–2018

	Reason for no cancer-directed surgical resection						*p*
	Surgery not recommended and not performed	Surgery not recommended, contraindicated due to comorbidities	Surgery recommended and performed	Surgery recommended but not performed, unknown reason	Surgery recommended but not performed, patient refused	Surgery recommended but not performed, as patient died	
	(*N* = 119487)	(*N* = 3827)	(*N* = 12014)	(*N* = 1469)	(*N* = 1178)	(*N* = 282)	
Age at diagnosis							<0.001
Mean (SD)	68.0 (12.0)	69.4 (11.7)	64.2 (13.8)	68.6 (12.0)	73.2 (13.1)	67.5 (12.2)	
Median [Min, Max]	68.0 [0, 100]	69.0 [20.0, 100]	65.0 [0, 100]	69.0 [21.0, 100]	75.0 [29.0, 100]	67.5 [23.0, 97.0]	
Sex							<0.001
Female	47978 (40.2%)	10516 (41.1%)	6008 (50.0%)	544 (37.0%)	659 (55.9%)	140 (49.6%)	
Male	71509 (59.8%)	15060 (58.9%)	6006 (50.0%)	925 (63.0%)	519 (44.1%)	142 (50.4%)	
Race							<0.001
White	94160 (78.8%)	21221 (83.0%)	9630 (80.2%)	1137 (77.4%)	904 (76.7%)	221 (78.4%)	
AI/AN	533 (0.4%)	138 (0.5%)	113 (0.4%)	13 (0.9%)	2 (0.2%)	0 (0.0%)	
Black	15418 (12.9%)	2547 (10.0%)	1488 (12.4%)	217 (14.8%)	190 (16.1%)	45 (16.0%)	
APAI	9045 (7.6%)	1670 (6.5%)	814 (6.8%)	97 (6.6%)	79 (6.7%)	16 (5.7%)	
Marital status							<0.001
Married	64394 (53.9%)	17920 (70.1%)	6882 (57.3%)	690 (47.0%)	419 (35.6%)	126 (44.7%)	
Nonmarried	55093 (46.1%)	7656 (29.9%)	5132 (42.7%)	779 (53.0%)	759 (64.4%)	156 (55.3%)	
Urban/rural							<0.001
Urban	104299 (87.3%)	3299 (86.2%)	10477 (87.2%)	1245 (84.8%)	1024 (86.9%)	246 (87.2%)	
Rural	15188 (12.7%)	528 (13.8%)	1537 (12.8%)	224 (15.2%)	154 (13.1%)	36 (12.8%)	
Yost Index							<0.001
Quintile 5	28900 (24.2%)	777 (20.3%)	3045 (25.3%)	249 (17.0%)	224 (19.0%)	72 (25.5%)	
Quintile 4	25953 (21.7%)	847 (22.1%)	2646 (22.0%)	279 (19.0%)	271 (23.0%)	53 (18.8%)	
Quintile 3	22127 (18.5%)	729 (19.0%)	2210 (18.4%)	262 (17.8%)	204 (17.3%)	51 (18.1%)	
Quintile 2	21033 (17.6%)	675 (17.67%)	2115 (17.6%)	308 (21.0%)	240 (20.4%)	48 (17.0%)	
Quintile 1	21474 (18.0%)	799 (20.9%)	1998 (16.6%)	371 (25.3%)	239 (20.3%)	58 (20.6%)	
Primary site							<0.001
Breast	13731 (11.5%)	432 (11.3%)	4302 (35.8%)	232 (15.8%)	364 (31.2%)	73 (25.9%)	
Colorectal	2619 (2.2%)	115 (3.0%)	848 (7.1%)	46 (3.1%)	66 (15.6%)	12 (4.3%)	
Esophagus	2333 (2.0%)	113 (3.0%)	29 (0.2%)	31 (2.1%)	14 (1.2%)	5 (1.8%)	
Kidney	4483 (3.8%)	225 (5.9%)	1733 (14.4%)	75 (5.1%)	90 (7.6%)	27 (9.6%)	
Liver	2949 (2.5%)	118 (3.1%)	72 (0.6%)	37 (2.5%)	16 (1.4%)	8 (2.8%)	
Lung	65919 (55.2%)	2158 (56.4%)	982 (8.2%)	646 (44.0%)	415 (35.2%)	129 (45.70%)	
Pancreas	3274 (2.7%)	126 (3.3%)	40 (0.3%)	29 (2.0%)	23 (2.0%)	7 (2.5%)	
Prostate	21405 (17.9%)	435 (11.4%)	2459 (20.5%)	338 (23.0%)	162 (13.8%)	11 (3.9%)	
Stomach	2129 (1.8%)	73 (1.9%)	82 (0.7%)	22 (1.5%)	10 (0.8%)	6 (2.1%)	
Urinary bladder	645 (0.5%)	32 (0.8%)	1467 (12.2%)	13 (0.9%)	15 (1.3%)	4 (1.4%)	

"Incidence—SEER Research Plus Data (Specialized with Census Tract SES/Rurality), 18 Regs (excluding AK), Nov 2020 Sub (2010–2018)" was used

#### Factors Associated with Refusing Surgical Treatment

Table 3 shows the direction, magnitude, and precision of the estimated associations between patient race and nSES with refusal of surgery both prior and after adjustment for clinical covariate confounders. Patients who are older at diagnosis were significantly more likely to have refused surgical treatment (aOR 1.07, 95% CI 1.06–1.07, per each additional year). nSES also was a persistent independent predictor of refusing surgical treatment even after adjustment for age, sex, race, marital status, urban/rural status, and primary cancer site. Patients with the lowest nSES had 57% higher odds of refusing surgical treatment compared with patients with the highest nSES (aOR 1.57, 95% CI 1.30–1.91, *p* < 0.001). Black patients were 34% more likely to refuse treatment compared with White patients (aOR 1.34, 95% CI 1.14–1.58, *p* = 0.003). Single patients were approximately twice as likely to refuse treatment compared with married patients (aOR 2.10, 95% CI 1.87–2.36, *p* < 0.001).

**Table 3:** Crude and adjusted odds ratios of refusing surgical treatment in patients with metastases to the bone and joints stratified by clinical covariates

	Odds ratio (95% CI)			*p*
	Crude	*p*	Adjusted	
Age at diagnosis		<0.001		<0.001
(Per additional year)	1.06 (1.05–1.06)		1.07 (1.06–1.07)	
Sex		<0.001		0.75
Female	1.00	1.00		
Male	0.79 (0.72–0.88)		1.03 (0.88–1.21)	
Race		0.22		
White	1.00		1.00	
AI/AN	0.41 (0.01–1.12)		0.73 (0.17–2.01)	0.67
Black	1.36 (1.18–1.56)		1.34 (1.14–1.58)	0.003
APAI	1.03 (0.84–1.26)		1.21 (0.96–1.51)	0.16
Marital status		<0.001		<0.001
Married	1.00		1.00	
Nonmarried	2.44 (2.20–2.71)		2.10 (1.87–2.36)	
Urban/rural status		0.77		0.25
Urban	1.00		1.00	
Rural	0.97 (0.84–1.13)		1.13 (0.95–1.34)	
Yost index		<0.001		<0.001
Quintile 5	1.00		1.00	
Quintile 4	1.39 (1.19–1.63)		1.35 (1.14–1.60)	
Quintile 3	1.25 (1.06–1.48)		1.26 (1.05–1.51)	
Quintile 2	1.55 (1.32–1.82)		1.59 (1.32–1.90)	
Quintile 1	1.63 (1.39–1.92)		1.57 (1.30–1.91)	
Primary site		0.01		
Breast	1.00		1.00	
Colorectal	0.91 (0.72–1.13)		0.71 (0.55–0.90)	0.02
Esophagus	5.68 (3.24–9.65)		6.98 (3.80–12.5)	<0.001
Kidney	0.61 (0.50–0.74)		0.69 (0.55–0.87)	0.008
Liver	2.62 (1.61–4.08)		2.67 (1.59–4.29)	<0.001
Lung	4.98 (4.37–5.69)		4.29 (3.65–5.05)	<0.001
Pancreas	6.77 (4.32–10.43)		6.15 (3.74–10.0)	<0.001
Prostate	0.78 (0.66–0.91)		0.45 (0.36–0.57)	<0.001
Stomach	1.43 (0.79–2.42)		1.31 (0.70–2.31)	0.45
Urinary Bladder	0.12 (0.08–0.18)		0.07 (0.05–0.11)	<0.001

"Incidence—SEER Research Plus Data (Specialized with Census Tract SES/Rurality), 18 Regs (excluding AK), Nov 2020 Sub (2010–2018)" was used

#### Race/Ethnicity, nSES, and Reasons for No-cancer-directed Surgical Resection

Table 4 shows the age-adjusted incidence rates of patients with metastatic cancer to the bone and joints stratified by Yost Index quintiles and their reasons for not undergoing surgical intervention. Patients in the lowest nSES quintile were most likely to not have surgery recommended (186.4 per 1 million, 95% CI 184.0–188.7; SE 1.2) compared with patients in the highest nSES quintile (142.1, 95% CI 140.6–143.7; SE 0.8). Patients in the lowest nSES quintile also had the highest rate of being contraindicated for surgery due to comorbid conditions (6.9, 95% CI 6.4–7.3; SE 0.2) compared with patients in the highest nSES quintile (3.7, 95% CI 3.4–3.9; SE 0.1). Patients in the lowest nSES quintile also were more likely to refuse offered surgery or not undergo a procedure for unknown reasons (2.2, 95% CI 2.0–2.5; SE 0.1 and 3.3, 95% CI 3.0–3.7; SE 0.2, respectively) than those in the highest nSES quintile (1.1, 95% CI 1.0–1.3; SE 0.1 and 1.2, 95% CI 1.1–1.4; SE 0.1, respectively).

**Table 4:** Age-adjusted incidence rates of patients living with metastatic cancer to the bone and joints, stratified by Yost Index Quintile and Reason for no cancer-directed surgical resection, 2010–2018

Surgery status	Yost index quintile				
	Quintile 1	Quintile 2	Quintile 3	Quintile 4	Quintile 5
	[95% CI]	[95% CI]	[95% CI]	[95% CI]	[95% CI]
Not recommended, not performed	186.4 [184.0–188.7] SE: 1.2	172.3 [170.2–174.5] SE: 1.1	164.5 [162.4–166.5] SE: 1.0	159.2 [157.4–161.1] SE: 0.9	142.1 [140.6–143.7] SE: 0.8
Not recommended, contraindicated	6.9 [6.4–7.3] SE: 0.2	5.5 [5.1–5.9] SE: 0.2	5.2 [4.8–5.6] SE: 0.2	5.0 [4.7–5.3] SE: 0.16	3.7 [3.4–3.9] SE: 0.1
Recommended and performed	19.9 [19.1–20.7] SE:0.4	20.3 [19.5–21.0] SE: 0.4	19.2 [18.5–19.9] SE: 0.4	19.1 [18.5–19.8] SE: 0.3	18.0 [17.4–18.6] SE: 0.28
Recommended, but not performed, unknown	3.3 [3.0–3.7] SE: 0.2	2.5 [2.2–2.7] SE: 0.1	2.0 [1.8–2.3] SE: 0.1	1.8 [1.6–2.0] SE: 0.1	1.2 [1.1–1.4] SE: 0.07
Recommended, but not performed, patient refused	2.2 [2.0–2.5] SE: 0.1	2.0 [2.2–2.7] SE: 0.1	1.6 [1.4–1.8] SE: 0.1	1.7 [1.5–1.9] SE: 0.1	1.1 [1.0–1.3] SE: 0.07
Recommended, but not performed, patient died	0.6 [0.4–0.7] SE: 0.1	0.5 [0.3–0.6] SE:0.1	0.6 [0.5–0.7] SE: 0.1	0.4 [0.3–0.5] SE: 0.04	0.4 [0.3–0.5] SE: 0.04

"Incidence—SEER Research Plus Data (Specialized with Census Tract SES/Rurality), 18 Regs (excluding AK), Nov 2020 Sub (2010–2018)" was used. 95% confidence intervals (CI) calculated using the Exact method. Standard Errors (SE) reported. All age-adjusted incidence rates are per 1,000,000 individuals in the population

Table 5 shows the age-adjusted incidence rates of patients living with metastatic cancer to the bone and joints stratified by race and ethnicity and their reasons for not undergoing surgery. Black patients were less likely to be recommended for surgery (186.4 per 1 million, 95% CI 184.0–188.7; SE 1.2) than White patients (142.1, 95% CI 140.6–143.7; SE 0.8). Black patients were also more likely to be contraindicated for surgery due to comorbid conditions (6.9, 95% CI 6.4–7.3; SE 0.2) compared with White patients (3.7, 95% CI 3.4–3.9; SE 0.1). Black patients were more likely to either refuse surgery or not have surgery for unknown reasons (2.2, 95% CI 2.0–2.5; SE 0.1 and 3.3, 95% CI 3.0–3.7; SE 0.2, respectively) compared with White patients (1.1, 95% CI 1.0–1.3; SE 0.1 and 1.2, 95% CI 1.1–1.4; SE 0.1, respectively). A first-order interaction between Race and the Yost Index was not statistically significant (*p* > 0.05)**,** indicating that race did not modify the effect of the Yost index on refusal of recommended surgical resection. Likewise, a first-order interaction between marital status was not statistically significant (*p* > 0.05), indicating that marital status did not modify the effect of the Yost index on refusal of recommended surgical resection.

**Table 5:** Age-adjusted incidence rates of patients living with metastatic cancer to the bone and joints, stratified by Race/Ethnicity and Reason for no cancer-directed surgical resection, 2010–2018

Surgery status	Race and ethnicity				
	NH-Black	NH-White	NH-AIAN	NH-API	Hispanic
	[95% CI]	[95% CI]	[95% CI]	[95% CI]	[95% CI]
Not recommended, not performed	213.4 [210.2–216.6] SE: 1.6	174.5 [173.4–175.6] SE: 0.6	137.5 [127.1–148.4] SE: 5.4	125.9 [123.5–128.1] SE: 1.2	123.0 [120.9–125.2] SE: 1.1
Not recommended, contraindicated	8.9 [8.3–9.6] SE: 0.3	5.6 [5.4–5.8] SE: 0.1	3.4 [2.0–5.5] SE: 0.8	2.7 [2.4–3.1] SE: 0.2	2.8 [2.5–3.2] SE: 0.2
Recommended and performed	22.6 [21.6–23.6] SE: 0.5	20.9 [20.5–21.3] SE: 0.2	19.2 [12.1–19.3] SE: 1.8	13.8 [13.0–14.6] SE: 0.4	16.0 [15.3–16.7] SE: 0.4
Recommended, but not performed, unknown	3.0 [2.6–3.4] SE: 0.2	2.1 [2.0–2.3] SE: 0.1	8.4 [5.8–11.6] SE: 1.4	1.6 [1.3–1.9] SE: 0.1	2.3 [2.0–2.6] SE: 0.2
Recommended, but not performed, patient refused	2.0 [2.2–2.7] SE: 0.2	1.8 [1.6–1.9] SE: 0.1	0.6 [0.1–1.8] SE: 0.4	1.2 [1.0–1.5] SE: 0.1	1.0 [0.8–1.3] SE: 0.1
Recommended, but not performed, patient died	0.7 [0.5–0.9] SE:0.1	0.5 [0.4–0.5] SE: 0.03	0.1 [0.0–0.9] SE: 0.1	0.3 [0.2–0.4] SE: 0.1	0.3 [0.2–0.4] SE: 0.1

"Incidence—SEER Research Plus Data (Specialized with Census Tract SES/Rurality), 18 Regs (excluding AK), Nov 2020 Sub (2010–2018)" was used. 95% confidence intervals calculated using the Exact method. All age-adjusted incidence rates are per 1,000,000 individuals in the population

*NH* non-Hispanic

## Discussion

Metastatic disease to bone has a poor prognosis and is a source of significant psychological and physical stress.^2^ The complex care and psychosocial needs of patients living with metastatic disease can modulate their indication for and acceptance of surgical intervention. The present work sought to understand whether socioeconomic factors were independently predictive of patient acceptance of offered surgery, finding that both race and socioeconomic status influenced the decision to undergo surgery. The decision to recommend surgery is influenced by performance status, systemic disease burden, mechanical stability, fracture risk, neurological risk, and life expectancy.^27,28^ Resection can be beneficial in patients with spinal metastases when surgery is expected to prolong life, and relieve symptoms, and in the combination with radiotherapy has become the standard therapy for eligible patients in the past decades.^29–31^ Although treatment is generally considered to be palliative, surgery aims to maintain spinal stability and increase quality of living.^32^ Multiple scores have been developed to estimated survival of patients and establish a cutoff for surgical referral in these patients; however, there is no universally recommended treatment strategy.^29^ Of patients with de novo metastatic cancer to the bone and joints from the top ten most prevalent primary cancer sites, only a minority of patients analyzed in our study were offered surgery (13.5%). Treatment options include en bloc spondylectomy, decompression of the spinal cord followed by stereotactic radiosurgery (“separation surgery”) and palliative surgery to ensure spinal stability via cement augmentation or release neurological symptoms via decompression.^29^ As advancements in the treatment of spinal tumors continue to progress and patients experience longer survival rates, surgical interventions are anticipated to play an increasingly significant role in the management of metastases. The present work reports that specific patient factors were predictive of refusing surgical treatment.

Older patients were more likely to refuse surgical treatment (aOR 1.07, 95% CI 1.06–1.07, per each additional year), which supports current literature that suggests that older patients are less likely to proceed with cancer treatment or surgical intervention.^33–36^ Several mechanisms may underlie elderly patient refusal of surgery, including the changing balance between the risks and benefits of surgery in an individual with a decreased potential lifespan that may lead to a greater emphasis on quality of life rather than lengthening survival.^34,37^ Older patients also may be in worse health and have significant comorbidities, which may impact their decision to proceed with invasive therapy.^36^

Our data support previous literature that marital status plays an important role in surgical utilization broadly and within oncology, with higher rates of refusing surgery among patients who were unmarried.^33–35^ This may underscore the need for financial, social, and physical support in driving patients to seek treatment and through the recovery process.^38^ Previous studies have reported that married couples share emotional burden, thus alleviating some of the cancer patient’s emotional distress and promoting the importance of survival.^39^ Our studied also showed that women were less likely to undergo surgery, which is consistent with the larger body of literature on female surgical utilization patterns.^33–35^ While causes of this trend have been proposed, to our knowledge no consensus has been achieved regarding this explain this utilization disparity, particularly in orthopedic surgery.^40^

Black patients were 35% more likely to refuse treatment than White patients. Previous works have highlighted differences in the personal influences on treatment discussions between races. Race and ethnicity dictated the final decision maker in the management of older breast cancer patients, with varying reliance on physician recommendations and family member input.^41^ A previous SEER study showed that not only are refusal rates higher among African Americans, these differences persisted, albeit narrowed, after adjusting for insurance type and marital status.^33^ While current studies have shown that the race gap in healthcare outcomes has decreased but remains associated with declining poverty levels in the Black and Hispanic populations,^42–46^ our study indicates that a substantial gap still persists in the patient’s decision to undergo surgery for metastatic bone disease. It is evident that while current interventions targeting outcome disparities between racial and ethnic populations may be effective, a focus should be placed on the barriers and personal factors that influence a patient’s decision to proceed with treatment.

nSES was a consistent independent predictor of refusing surgical treatment, even when controlling for covariates. Patients in the lowest nSES quintile were less likely to have surgery recommended to them and were more likely to be contraindicated for surgery because of comorbid conditions. Patients in the lowest nSES quintile also were 57% more likely to refuse surgery or not undergo surgery for unknown reasons (aOR 1.57, 95% CI 1.30–1.91, *p* < 0.001). While this has not been previously reported in orthopedic procedures, there is mixed oncologic data concerning nSES and the decision to undergo surgery. One previous study found that low neighborhood socioeconomic status was associated with increased surgery utilization among metastatic breast cancer patients.^47^ Another study found that low nSES patients less likely to go undergo surgery for pancreatic cancer.^48^ Factors closely associated with nSES, such as social and financial support, type of work, and non-English first language, may act as barriers and additive influences on patient decision-making for surgical intervention.^49^ Despite this understanding, there is limited literature on the main drivers of how nSES affects patient decision-making in the setting of metastatic disease, especially to bone. The present work suggests that the variables used in the Yost Index, such as income, class, and household characteristics, individually or synergistically contribute to patient decision-making.

The Yost Index served as a measure of neighborhood socioeconomic status (nSES) in this study, which encompassed employment, income, living conditions, infrastructure, education, and the social environment. These characteristics have individually shown to influence patient care and outcomes.^50–52^ A comprehensive review conducted by Sorice et al.^53^ compared indices of nSES and found that the Yost-Index was a reliable tool to assess patients’ vulnerability. Living in deprived neighborhoods has been linked to various health risks, including higher air pollution,^54^ decreased quality of sleep,^55^ and inflammation.^56^ Living in a safe, walkable environment with low crime rates and desirable recreational areas, such as parks, promotes physical and mental health.^57–60^ Lower neighborhood socioeconomic status has been linked to increased mortality risk.^61,62^ There is strong evidence in literature that moving from high-poverty to lower-poverty neighborhoods can have a positive long-term effect on mental and physical well-being.^63^ Lower nSES also has been associated with decreased mental health and reduced cognitive function.^57,64^ Residents of affluent neighborhoods have been linked to more favorable dietary patterns^65,66^ and demonstrated a reduced likelihood of enga/ging in alcoholism and smoking.^67^ They also have been shown to exhibit a higher capacity to quit smoking^68^ and a better adherence to prescription medications and adherence to cancer screening.^69,70^ Nouri et al.^71^ found that documentation of advanced healthcare planning was significantly inferior in patients with lower nSES compared with individuals living in more affluent areas, even after adjusting for amount of care encounters. While education and language barrier can affect patients understanding of treatment instructions and planning, there is a lack of understanding of healthcare providers which factors influence health-seeking behaviors.^72^ Social beliefs highly influence patients’ interactions with medical providers and racial minorities were shown to exhibit lower level of trust in the healthcare system with higher rates of refusal of care.^73,74^ Jakobsen et al.^75^ found that residents of deprived neighborhoods exhibited a lack of trust which could negatively influence engagement with health care providers.

However, it remains unclear to which extend these factors interfere with decision making and refusal of surgery. We analyzed patients that had already encountered healthcare providers and were recommended for surgery; thus, access to health care alone is unlikely to explain our findings. The link of nSES with refusal of surgical care specifically indicates a deeper systematic discrepancy, that surprisingly persisted independently of race or ethnicity. Identifying risk groups is crucial for providing universal health standards and understanding factors that affecting decision making and clinical outcomes in patients. For instance, patients in rural areas may face limited access to high-capacity healthcare facilities, leading to less favorable treatment responses.^76,77^ Insufficient access to medical care centers complicates follow-up and postsurgical rehabilitation, with neighborhood socioeconomic status impacting recovery in stroke survivors.^76,78^ Moreover, zip code areas have been shown to affect care fragmentation, influencing outcomes in cancer patients.^79^ As elaborated, neighborhood and living environment have been shown to effect mental health and belief systems that are highly likely to influence social interaction and health seeking behavior. However, it remains unclear to which extend these socioeconomic factors are associated with decision making, and our study aimed to gain novel insight into understanding that relationship by analyzing refusal of surgical management and neighborhood socioeconomic factors encompassed by the Yost index.

There are several strengths to this study. The study population from this database represents the racial and ethnic heterogeneity of the United States. The database includes data from 18 national cancer registries from geographically, racially, and ethnically diverse U.S. regions.^80^ A standardized approach for calculating Yost Index quintiles for census tracts across the entire United States was utilized in making nSES comparisons. In standardizing different states and regions, across measures of economic development, population density, race, and other contributing factors, the authors could make direct nSES comparisons. Validated studies have shown that nSES is better quantified by census tract variables than county-level variables^22^ due to the smaller size and decreased economic diversity of census tracts. Finally, this work is novel in its comprehensive approach to metastatic disease to bone, including 13 ICD-0-3 codes and a majority of the bones of the human body.

There are several limitations to this work beyond those intrinsic to any retrospective database study. First, data collection on insurance status might serve as a confounding variable within this study. The SEER Census Tract-level nSES and Rurality Database that was used in this study was not compatible with SEER-Medicare data, so it was not possible to further assess insurance status. Another limitation of this study was that due to inconsistencies in merging the 7th and 8th AJCC editions, which covered the span of the study period, we did not include tumor T and N stage in this analysis. Given the study population was all metastatic at diagnosis, AJCC M stage and stratification by primary cancer subtype likely has a larger impact than T and N stage on mortality. Last, misclassification of cancer-related deaths is possible, as local or regional relapses may be incorrectly classified as a new cancer or metastasis to an adjacent organ may be misattributed.

## Data Availability

This cross-sectional study utilized data from the publicly accessible Surveillance, Epidemiology, and End Results (SEER) database of the National Cancer Institute (https://seer.cancer.gov/). Because the data is publicly available, no institutional review board review was necessary. This study followed the Strengthening the Reporting of Observational Studies in Epidemiology (STROBE) reporting guidelines.
